# Biparametric Versus Multiparametric MRI for VI-RADS Assessment: Reproducibility Relative to Routine mpMRI Reporting and Impact of Radiologist Experience in a Single-Center Study

**DOI:** 10.3390/cancers18060999

**Published:** 2026-03-19

**Authors:** Fabrizio Urraro, Nicoletta Giordano, Vittorio Patanè, Maria Chiara Brunese, Claudia Rossi, Antonio Cioffi, Anna Russo, Carlo Varelli, Fiammetta Cappabianca, Alfonso Reginelli

**Affiliations:** 1 Department of Life Sciences, Health and Health Professions, Link Campus University, 00165 Rome, Italy; f.urraro@unilink.it; 2 Department of Precision Medicine, University of Campania “Luigi Vanvitelli”, Piazza Luigi Miraglia 2, 80138 Naples, Italy; 3 Radiology Department, CTO Hospital, AORN dei Colli, 80131 Naples, Italy; 4 Department of Urology, Ospedale del Mare, 80147 Napoli, Italy; 5 Radiology Unit, Istituto Diagnostico Varelli, 80126 Naples, Italy; 6 School of Medicine, Università Campus Bio-Medico di Roma, 00128 Rome, Italy

**Keywords:** bladder cancer, magnetic resonance imaging, VI-RADS, biparametric MRI, multiparametric MRI, radiologist experience, tumor staging, muscle-invasive bladder cancer

## Abstract

**Background**: We tested whether a contrast-free protocol can reproduce contrast-enhanced VI-RADS scoring and whether reader expertise influences results. **Methods**: In this retrospective single-center study (January–December 2024), 65 patients (69 lesions) underwent bladder multiparametric MRI. Two blinded radiologists assigned VI-RADS scores using only T2-weighted and diffusion-weighted imaging (biparametric, non-contrast MRI): an expert (>15 years in urogenital radiology) in genitourinary MRI and a non-expert (5 years of experience in genitorurinary radiology). Two complementary reference standards were used. For reproducibility analysis, the reference standard was the VI-RADS score from the original clinical report based on the full multiparametric examination including contrast-enhanced imaging. For diagnostic accuracy analysis, histopathology was used as the reference standard for muscle-invasive versus non-muscle-invasive disease. Agreement was evaluated with confusion matrices, overall agreement, and weighted Cohen’s kappa. Discrimination for high likelihood of muscle invasion (VI-RADS ≥ 4) was assessed with receiver operating characteristic analysis. **Results**: Reference scores were VI-RADS 2 (34.8%), 3 (14.5%), 4 (20.3%), and 5 (30.4%). Agreement was higher for the expert than the non-expert (73.9% vs. 56.5%; weighted kappa 0.74 [95% confidence interval 0.56–0.89] vs. 0.58 [0.37–0.75]). The area under the curve for VI-RADS ≥ 4 was 0.87 (0.78–0.95) for the expert and 0.81 (0.69–0.91) for the non-expert. Sensitivity at a biparametric threshold of VI-RADS ≥ 4 was 88.6% for both readers; specificity was 85.3% vs. 73.5%. Post-resection cases showed more discrepancies, mainly overstaging. **Conclusions**: Contrast-free biparametric MRI may approximate multiparametric VI-RADS scoring only in treatment-naïve pre-TURBT cases with clearly low-risk, non-equivocal imaging features, but performance is reader-dependent and less reliable in equivocal, higher-risk, and post-resection examinations. Contrast-enhanced multiparametric MRI remains preferred for staging.

## Simple Summary

Bladder cancer treatment depends heavily on whether the tumor is still limited to its muscle invasiveness playing a pivotal role in its management. Doctors often use magnetic resonance imaging to estimate the chance of muscle invasion, but the full examination usually includes an injected contrast agent, which adds time and cost and cannot be given to some patients. This study tested whether a simpler, contrast-free magnetic resonance imaging approach can provide the same practical risk grading as the standard contrast-enhanced examination in routine care. The authors compared scores from two readers with different levels of experience and examined where and why disagreements occur, including after prior tumor removal surgery. The results suggest that contrast-free imaging may be acceptable only in carefully selected pre-TURBT cases with clearly low-risk, non-equivocal imaging features when interpreted by experienced radiologists, whereas standard contrast-enhanced imaging remains important for equivocal, higher-risk, and post-surgery settings.

## 1. Introduction

Bladder cancer is among the most common malignancies of the urinary tract and remains a major global health burden [1,2,3,4,5]. Clinical management is highly dependent on accurate local staging, as treatment strategies and outcomes differ substantially between non–muscle-invasive bladder cancer (NMIBC) and muscle-invasive bladder cancer (MIBC) [6,7,8,9]. NMIBC is usually managed with transurethral resection of bladder tumor (TURBT), followed by intravesical therapy and surveillance, whereas MIBC more often requires radical treatment, including radical cystectomy, neoadjuvant chemotherapy, or bladder-preserving multimodal approaches [10,11]. Consequently, reliable pre-treatment identification of tumor invasion into the muscularis propria is a key step in bladder cancer workup.

Conventional staging based on cystoscopy and TURBT has well-known limitations [12,13,14,15]. Sampling error, intratumoral heterogeneity, and interobserver variability may impair staging reliability, particularly when differentiating high-grade T1 disease from early muscle invasion [16]. These limitations have prompted increasing interest in non-invasive imaging tools to support local staging. Magnetic resonance imaging (MRI) has emerged as a valuable adjunct due to its high soft-tissue contrast and functional imaging capabilities, which allow visualization of the layered bladder wall architecture and assessment of tumor extension beyond what may be captured by endoscopic evaluation alone [17,18,19]. To standardize acquisition and interpretation, the Vesical Imaging–Reporting and Data System (VI-RADS) was introduced in 2018 [20,21,22,23,24]. VI-RADS provides a structured framework to estimate the likelihood of muscle invasion using multiparametric MRI (mpMRI), combining anatomical information from T2-weighted imaging (T2WI) with functional assessment from diffusion-weighted imaging (DWI) and dynamic contrast-enhanced (DCE) imaging [16,24,25,26,27,28,29,30]. Since its introduction, VI-RADS has been widely validated and has shown high performance for identifying muscle-invasive disease, as well as improved interobserver agreement compared with non-standardized MRI interpretation.

Despite these advantages, mpMRI entails practical constraints that may limit its broader implementation. DCE acquisition increases examination time and costs and requires intravenous administration of gadolinium-based contrast agents [31,32,33,34]. Contrast administration may be contraindicated in patients with renal impairment or prior hypersensitivity reactions and has raised additional concerns regarding gadolinium retention [35,36,37]. In this context, simplified MRI protocols have been proposed to reduce complexity while maintaining clinically useful risk stratification. Biparametric MRI (bpMRI), based on T2WI and DWI while omitting DCE, offers potential logistical benefits, including shorter scan time and avoidance of contrast administration [38,39,40]. Several studies have explored bpMRI-based VI-RADS assessment, with some reporting performance comparable to mpMRI for detecting muscle-invasive disease in selected cohorts [41,42,43,44,45]. In parallel, modified approaches that downplay or exclude contrast-enhanced imaging have further supported the rationale for contrast-free MRI pathways in bladder cancer staging [9,46,47].

However, evidence remains heterogeneous and context-dependent. Reported bpMRI performance varies across studies, likely reflecting differences in patient selection, imaging protocols, reference standards, and reader expertise. Importantly, the incremental value of DCE imaging may not be uniform across clinical scenarios. While contrast enhancement may add limited information in clearly low-risk lesions, it may be more relevant in intermediate- and high-risk cases, where accurate delineation of the tumor–muscle interface is critical. Moreover, the post-treatment setting introduces additional challenges. After TURBT, inflammation, edema, and fibrosis can alter bladder wall morphology and diffusion signal, potentially mimicking residual or recurrent tumor on T2WI and DWI alone; omission of DCE may therefore increase interpretative uncertainty and misclassification risk [48,49,50].

Another issue that warrants further investigation is the influence of radiologist expertise on bpMRI interpretation. Reader experience affects VI-RADS performance in mpMRI-based evaluations, but fewer studies have specifically addressed how removing DCE impacts readers with different levels of experience [51,52,53,54]. It is plausible that bpMRI, by relying more heavily on subtle morphological and diffusion-weighted cues, may amplify experience-related variability. Furthermore, although histopathology remains the definitive reference standard, everyday clinical decision-making often relies on mpMRI-based VI-RADS assessment to guide multidisciplinary discussions and treatment planning. Whether bpMRI can reliably reproduce this real-world, mpMRI-driven risk stratification has not been fully clarified.

Against this background, the aims of the present study were twofold: first, to evaluate whether bpMRI-based VI-RADS assessment can reproduce routine mpMRI-based VI-RADS categorization in a contemporary single-center cohort; and second, to assess the diagnostic performance of bpMRI for differentiating muscle-invasive from non-muscle-invasive bladder cancer using histopathology as the reference standard. Particular attention was paid to the impact of radiologist expertise, patterns of misclassification, and clinically relevant thresholds, including VI-RADS ≥ 4. By combining agreement analysis, confusion matrix evaluation, weighted kappa statistics, ROC-based performance metrics, and histopathological correlation, we sought to better define the potential role—and limitations—of bpMRI as a contrast-free approach for VI-RADS-based bladder cancer assessment.

## 2. Methods

### 2.1. Study Design and Population

This retrospective, single-center observational study was conducted at University Hospital “Luigi Vanvitelli”, Naples, Italy. The study was conducted in accordance with the Declaration of Helsinki and received approval from the local ethics committee (Prot. 154/i/2026, 5 January 2026). Written informed consent was obtained from all patients for enrollment and for potential publication of anonymized data and images.

Consecutive patients who underwent bladder multiparametric MRI (mpMRI) for suspected or known bladder cancer between January 2024 and December 2024 were screened for eligibility. According to the original VI-RADS concept and subsequent literature, bladder MRI is optimally performed before TURBT for primary local staging; when performed after TURBT or intravesical treatment, interpretation may be affected by post-procedural changes and should therefore be considered with caution [55,56].

Patients were included if a complete bladder mpMRI examination was available, including T2-weighted imaging (T2WI), diffusion-weighted imaging (DWI), and dynamic contrast-enhanced (DCE) sequences, and if at least one bladder lesion was assessable according to VI-RADS criteria. Patients were excluded in case of non-diagnostic image quality or incomplete MRI protocol.

A total of 65 patients met the inclusion criteria and constituted the final study cohort. Overall, 69 lesions were included in the analysis. Sixty-one patients had one assessable lesion, whereas four patients had two assessable lesions. The population included 47 men and 18 women, with an age range of 65–83 years. Fifty-six patients underwent MRI in the pre-treatment setting, whereas nine patients had undergone transurethral resection of bladder tumor (TURBT) prior to MRI acquisition and therefore represented a post-treatment subgroup. The study was designed to simulate a real-world clinical scenario in which a bpMRI-based workflow is considered as an alternative to standard mpMRI for VI-RADS-based staging, while acknowledging that VI-RADS is most firmly established for pre-TURBT local staging and that post-TURBT assessment is intrinsically more challenging [57,58].

Given the lesion-based design, observations are not strictly independent in patients with multiple lesions. However, clustering was limited, since only four patients contributed two lesions, while all others contributed a single lesion. Although each lesion was evaluated separately, some within-patient correlation cannot be excluded because lesions from the same patient share the same imaging examination context. Therefore, analyses should be regarded as primarily exploratory and focused on clinically meaningful effect sizes, agreement patterns, and misclassification trends.

### 2.2. MRI Acquisition Protocol

MRI was performed on a 1.5-T system (Signa Voyager, GE Healthcare, Milwaukee, WI, USA). All MRI examinations were performed in routine clinical practice using a standardized bladder mpMRI protocol compliant with VI-RADS recommendations. The protocol included high-resolution multiplanar T2-weighted imaging for anatomical assessment of bladder wall layers and tumor morphology, diffusion-weighted imaging (DWI) with corresponding apparent diffusion coefficient (ADC) maps for functional assessment, and dynamic contrast-enhanced (DCE) imaging after intravenous administration of gadobutrol at a standard dose of 0.1 mmol/kg (Bayer SpA, Milan, Italy).

The T2-weighted protocol included axial T2 PROPELLER fat-suppressed imaging (TR 9041.0 ms, TE 149.7 ms, slice thickness 1.5 mm), sagittal T2 PROPELLER fat-suppressed imaging (TR 8196 ms, TE 140.3 ms, slice thickness 1.5 mm), axial T2 PROPELLER imaging without fat suppression (TR 10,509 ms, TE 162.3 ms, slice thickness 1.5 mm), and coronal T2 PROPELLER fat-suppressed imaging (TR 9000 ms, TE 164.2 ms, slice thickness 1.5 mm). DWI was acquired using a free-breathing spin-echo echo-planar imaging sequence with spectral fat saturation, with b-values of 50, 1000, and 1500 s/mm^2^. DCE imaging was performed using a T1-weighted gradient-echo sequence (TR 5.9 ms, TE 3.1 ms, slice thickness 1.5 mm) after contrast injection at a rate of 1.5–2.0 mL/s, with six acquisitions obtained at a temporal resolution of 30 s.

To optimize bladder distension, patients were instructed to drink approximately 500 mL of water prior to the examination. In our routine clinical practice, an antiperistaltic agent is not routinely administered for bladder MRI, as satisfactory image quality is generally achieved without mandatory use of antispasmodic medication. For the purposes of this study, only T2WI and DWI/ADC sequences were extracted from the full mpMRI dataset and provided to the readers to simulate a biparametric MRI (bpMRI) examination, in accordance with published non-contrast VI-RADS concepts.

### 2.3. Image Interpretation

Two radiologists independently reviewed the bpMRI-only datasets. The two readers were different from the radiologist who had originally reported the routine clinical mpMRI examinations used as the reference standard. To reduce potential confirmation bias, bpMRI datasets were anonymized and presented in randomized order for independent review. Reader 1 was a radiologist with dedicated experience in genitourinary MRI and routine involvement in bladder mpMRI interpretation and VI-RADS reporting, whereas Reader 2 was a radiologist without dedicated subspecialty training in genitourinary imaging.

Both readers were blinded to clinical information, histopathological findings, the original mpMRI reports, and each other’s evaluations. Each reader assigned a bpMRI-based VI-RADS score ranging from 1 to 5 for each lesion, following the published VI-RADS criteria and recommendations using only T2WI and DWI/ADC information. Readers did not have access to DCE images, thereby replicating a bpMRI-only interpretation workflow.

### 2.4. Reference Standard

Two complementary reference standards were used according to the two study objectives. For the reproducibility analysis of bpMRI-based VI-RADS categorization, the reference standard was the VI-RADS score reported in the original clinical mpMRI report, which was based on the full multiparametric dataset including T2WI, DWI/ADC, and DCE sequences. These reports were generated during routine clinical practice by an experienced genitourinary radiologist and represented the imaging-based risk stratification used for patient management. This imaging-based reference standard was selected because the specific aim of this analysis was to determine whether a contrast-free abbreviated protocol could reproduce the standard mpMRI-derived VI-RADS assessment used in daily practice. Histopathology cannot serve as a direct reference for full ordinal VI-RADS reproducibility because pathological staging does not correspond one-to-one to the five-category imaging score.

For the diagnostic accuracy analysis, histopathology was used as the reference standard for classification of muscle-invasive versus non-muscle-invasive disease. Histopathological results were obtained from TURBT, repeat resection, and/or cystectomy specimens according to routine clinical management. For this binary endpoint, VI-RADS scores were dichotomized using the clinically relevant threshold of VI-RADS ≥ 4 to indicate a high likelihood of muscle invasion.

### 2.5. Statistical Analysis

Statistical analyses were performed to evaluate agreement between bpMRI-based VI-RADS assessment and routine mpMRI-based VI-RADS reporting, to characterize misclassification patterns, and to assess discrimination at a clinically relevant threshold. Given the retrospective design, analyses were intended to assess agreement and discrimination in a real-world reading setting rather than within a prospective randomized paired-reading framework. VI-RADS score distributions were summarized using counts and percentages. Agreement between bpMRI scores and the reference mpMRI scores was assessed using category-specific agreement rates and confusion matrix analysis to describe the direction and magnitude of discrepancies.

Given the ordinal nature of VI-RADS, weighted Cohen’s kappa (κ) with quadratic weights was calculated to quantify agreement beyond chance, penalizing larger category differences more heavily than minor shifts. To evaluate discrimination for a clinically relevant binary stratification, receiver operating characteristic (ROC) analysis was performed using VI-RADS ≥ 4 as the threshold indicating a high likelihood of muscle invasion according to the reference mpMRI report. The area under the ROC curve (AUC) was calculated separately for the expert and non-expert readers. Sensitivity, specificity, and false-positive and false-negative patterns at the VI-RADS ≥ 4 threshold were derived from the confusion matrices to support interpretation of ROC results.

To provide uncertainty estimates, 95% confidence intervals for AUC and weighted κ were obtained using bootstrap resampling. For the diagnostic accuracy analysis against histopathology, bpMRI VI-RADS scores were dichotomized using the threshold of VI-RADS ≥ 4 to identify lesions at high likelihood of muscle invasion. Sensitivity, specificity, positive predictive value, negative predictive value, and overall accuracy were calculated separately for the expert and non-expert readers. Where appropriate, the diagnostic performance of routine mpMRI VI-RADS assessment was also compared with histopathology for contextual interpretation. Given the exploratory nature of subgroup analyses and the limited number of post-TURBT examinations, results in the post-TURBT subgroup were analyzed descriptively.

## 3. Results

### 3.1. Patients’ Characteristics

**Figure 1:**
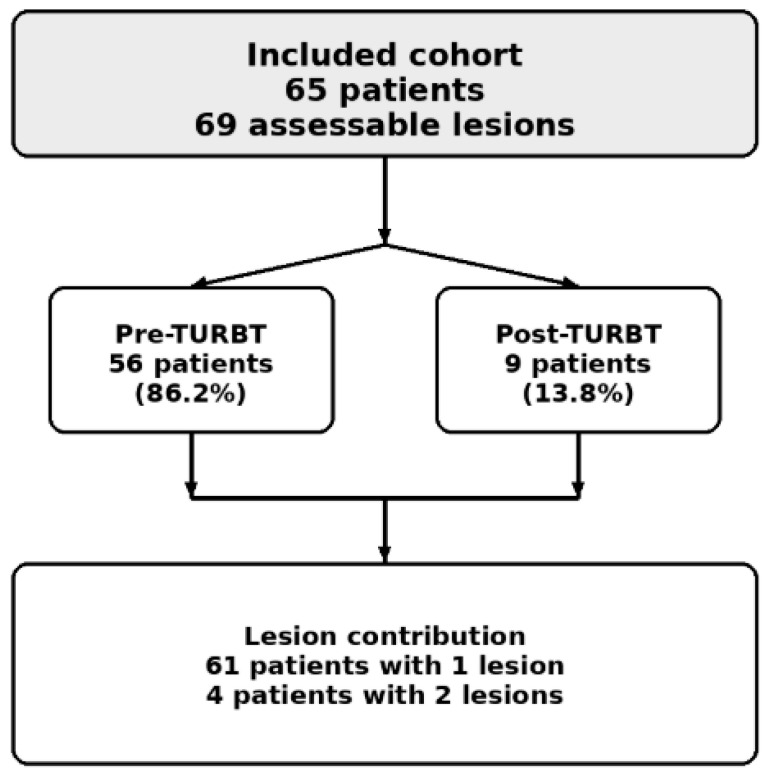
Overview of the study cohort. Study cohort overview showing 65 included patients, 69 assessable lesions, 56 pre-TURBT patients, and 9 post-TURBT patients. Among the included patients, 61 had one assessable lesion and 4 had two assessable lesions.

The final study population consisted of 65 patients who underwent bladder mpMRI during the study period, contributing 69 assessable lesions. Of these, 61 patients had a single assessable lesion and 4 patients had two assessable lesions. Most patients were male (47/65, 72.3%), while 18 were female (27.7%). Patient age ranged from 65 to 83 years. Nine patients (13.8%) had previously undergone transurethral resection of bladder tumor (TURBT) prior to MRI examination, constituting a clinically relevant post-treatment subgroup. The remaining 56 patients (86.2%) underwent MRI before resection, representing the main pre-treatment cohort for VI-RADS-based local staging. Patient demographics and baseline clinical characteristics are summarized in Table 1. An overview of the study cohort is provided in Figure 1.

### 3.2. Distribution of Reference mpMRI VI-RADS Scores

**Figure 2:**
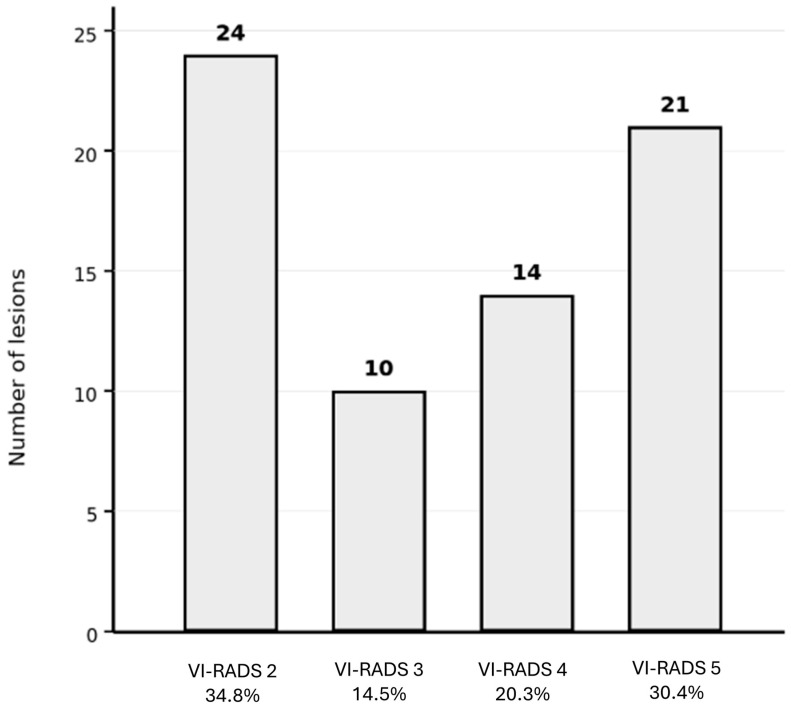
Distribution of reference mpMRI VI-RADS scores. Bar chart showing the distribution of reference mpMRI VI-RADS categories across the 69 assessed lesions: VI-RADS 2, n = 24 (34.8%); VI-RADS 3, n = 10 (14.5%); VI-RADS 4, n = 14 (20.3%); and VI-RADS 5, n = 21 (30.4%).

According to the original clinical mpMRI reports used as the reference standard, lesions were classified as VI-RADS 2 in 24/69 cases (34.8%), VI-RADS 3 in 10/69 cases (14.5%), VI-RADS 4 in 14/69 cases (20.3%), and VI-RADS 5 in 21/69 cases (30.4%). This distribution reflects a cohort enriched in intermediate- and high-risk lesions, consistent with a tertiary referral oncologic population (Figure 2).

**Figure 3:**
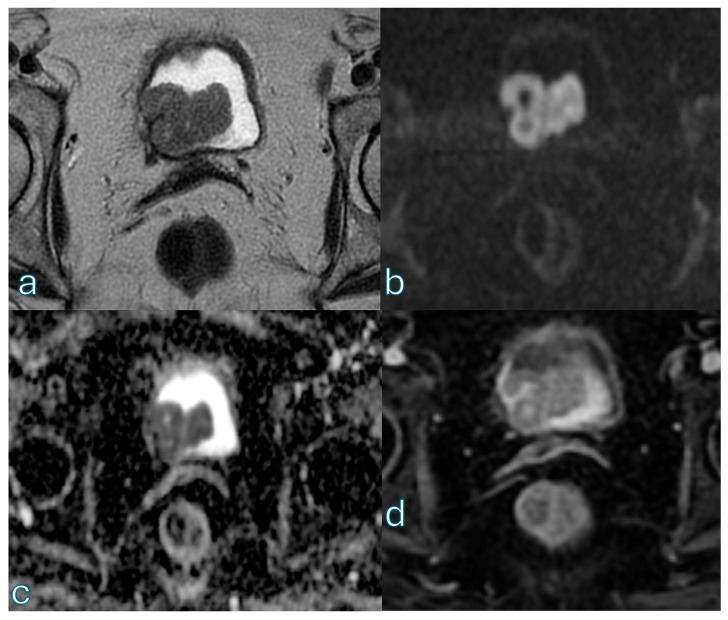
Axial T2-weighted imaging (T2WI) (**a**) demonstrates a pedunculated exophytic lesion arising from the right lateral bladder wall, measuring approximately 3 cm in maximum diameter, DCE provides temporal information on lesion, a feature that could suggest a higher VI-RADS category based on size alone. On diffusion-weighted imaging (DWI) (**b**), the lesion shows the characteristic inchworm sign, with curvilinear diffusion hyperintensity confined to the mucosal layer and corresponding signal on the ADC (**c**) map, already orienting the assessment toward a VI-RADS 2 category. DCE (**d**) imaging confirms preservation of the muscularis propria without evidence of early tumoral infiltration. Overall, despite its size, the combined imaging findings are consistent with a VI-RADS score of 2, highlighting the pivotal role of diffusion-weighted imaging in lesion risk stratification.

An illustrative case showing how diffusion-weighted imaging can support low-risk categorization despite lesion size is provided in Figure 3.

### 3.3. bpMRI Performance: Expert Reader

For the expert reader, bpMRI-based VI-RADS assessment showed substantial concordance with the reference mpMRI classification. Among lesions classified as VI-RADS 2 on mpMRI (n = 24), 21/24 (87.5%) were correctly assigned on bpMRI, with the remaining three cases overstaged to VI-RADS 5. For mpMRI VI-RADS 3 lesions (n = 10), 7/10 (70.0%) were correctly classified, with one lesion downgraded to VI-RADS 2 and two upgraded to VI-RADS 4. For mpMRI VI-RADS 4 lesions (n = 14), 8/14 (57.1%) were correctly classified, while the remaining lesions were distributed across adjacent categories. For mpMRI VI-RADS 5 lesions (n = 21), 15/21 (71.4%) were correctly classified, with most errors consisting of downgrading to VI-RADS 4. The complete confusion matrix for the expert reader is reported in Table 2.

### 3.4. bpMRI Performance: Non-Expert Reader

bpMRI interpretation by the non-expert reader showed lower agreement with the reference mpMRI report and broader misclassification patterns. Among mpMRI VI-RADS 2 lesions (n = 24), 17/24 (70.8%) were correctly classified, with frequent overstaging, particularly to VI-RADS 5. Among mpMRI VI-RADS 3 lesions (n = 10), 5/10 (50.0%) were correctly classified, with both understaging and overstaging observed. Among mpMRI VI-RADS 4 lesions (n = 14), 6/14 (42.9%) were correctly classified, with many lesions overstaged to VI-RADS 5. Among mpMRI VI-RADS 5 lesions (n = 21), 11/21 (52.4%) were correctly classified, and the most common error was downgrading to VI-RADS 4. The complete confusion matrix for the non-expert reader is reported in Table 3.

### 3.5. Agreement Analysis

**Figure 4:**
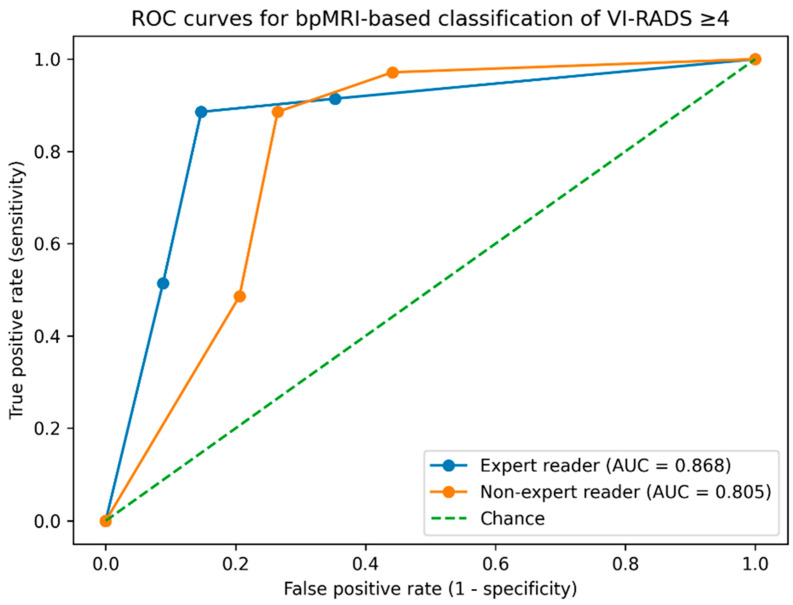
Receiver operating characteristic (ROC) curves for bpMRI-based classification of VI-RADS ≥ 4 using the reference mpMRI report as the standard. Curves are shown for the expert and non-expert readers; AUC values are derived from the bpMRI VI-RADS ordinal scores.

Weighted Cohen’s kappa showed higher agreement between bpMRI and mpMRI for the expert reader than for the non-expert reader. For the expert reader, weighted κ was 0.74 (95% CI: 0.56–0.89), whereas for the non-expert reader weighted κ was 0.58 (95% CI: 0.37–0.75). Overall categorical agreement was 51/69 (73.9%) for the expert reader and 39/69 (56.5%) for the non-expert reader. A summary of agreement and discrimination metrics is reported in Table 4. To improve interpretation of disagreement patterns, stratified disagreement rates by reference risk category (VI-RADS 2–3 vs. 4–5) and by clinical setting (post-TURBT vs. non-TURBT) are shown in Figure 4.

### 3.6. ROC Analysis for High-Likelihood Muscle Invasion (VI-RADS ≥ 4)

ROC analysis was performed using VI-RADS ≥ 4 as a clinically relevant threshold indicating a high likelihood of muscle invasion according to the reference mpMRI report. Using bpMRI-based VI-RADS as the ordinal classifier, the expert reader achieved an AUC of 0.868, whereas the non-expert reader achieved an AUC of 0.805. At the bpMRI threshold of VI-RADS ≥ 4, sensitivity was 88.6% for both readers, while specificity was higher for the expert reader (85.3%) than for the non-expert reader (73.5%), consistent with more frequent overstaging by the non-expert reader. Given that 34/69 lesions in the study cohort were classified as VI-RADS < 4 by the reference mpMRI report, these specificity values correspond to approximately 7 false-positive classifications per 100 similar cases for the expert reader and 13 per 100 similar cases for the non-expert reader. The ROC curves for both readers are shown in Figure 4.

### 3.7. Histopathological Correlation for Muscle-Invasive Disease

Histopathology represents the biological reference standard for differentiating muscle-invasive from non-muscle-invasive bladder cancer. In the present study, histopathological findings were available within routine clinical management and were considered in the interpretation of the binary endpoint of muscle invasion. Overall, the imaging-based results were consistent with good concordance for identifying lesions at high likelihood of muscle invasion, supporting the clinical validity of the bpMRI approach, although the primary focus of the present analysis remained the comparison between bpMRI-based and routine mpMRI-based VI-RADS assessments. Because histopathological sampling was not uniformly structured for a lesion-by-lesion five-category VI-RADS comparison, pathological data were not used as the reference standard for ordinal agreement analyses.

### 3.8. Post-TURBT Subgroup Analysis

**Figure 5:**
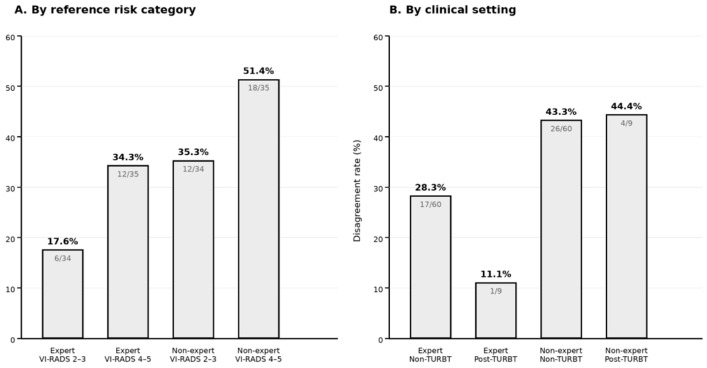
Stratified disagreement rates between bpMRI-based and reference mpMRI-based VI-RADS assessments. Disagreement rates are shown separately for the expert and non-expert readers according to reference risk category (VI-RADS 2–3 vs. VI-RADS 4–5) and clinical setting (non-TURBT vs. post-TURBT). Discordance was higher in high-risk than in low-risk lesions for both readers. In the post-TURBT setting, disagreement appeared more pronounced for the non-expert reader, whereas interpretation for the expert reader is limited by the small subgroup size.

Among the nine post-TURBT patients, disagreement patterns differed between readers. For the expert reader, 8/9 cases were concordant, with 1 overstaged case. For the non-expert reader, 5/9 cases were concordant, with 3 overstaged cases and 1 understaged case. Given the very small size of this subgroup, these findings should be interpreted descriptively (Figure 5).

**Figure 6:**
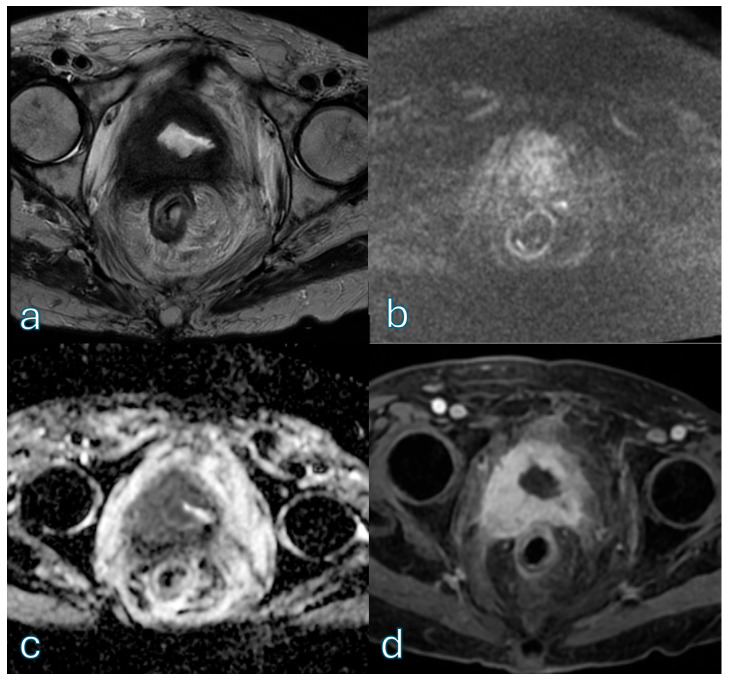
Multiparametric MRI demonstrates a post-TURBT bladder with intraluminal blood products, resulting in marked bladder wall irregularity and thickening. On T2-weighted imaging (**a**), postoperative changes and hemorrhagic content obscure normal wall stratification, a pattern that could lead to misclassification as VI-RADS 5 if assessed without contrast enhancement. However, diffusion-weighted imaging/ADC maps (**b**,**c**) do not show convincing focal diffusion restriction suggestive of viable tumor infiltration. Importantly, dynamic contrast-enhanced (**d**) imaging demonstrates absence of early tumoral enhancement and preservation of the muscular layer, allowing correct interpretation of postoperative changes rather than muscle-invasive disease. This case highlights the critical role of multiparametric MRI in the post-TURBT setting and the limitations of bpMRI-only assessment, where the risk of clinically relevant overstaging is substantial.

An illustrative post-TURBT case in which contrast-enhanced imaging prevents clinically relevant overstaging is shown in Figure 6. A summary of agreement patterns in the post-TURBT subgroup is reported in Table 5.

Misclassifications predominantly involved overstaging on bpMRI, consistent with post-procedural inflammatory or fibrotic changes that may mimic tumor-related wall abnormalities on T2WI and DWI/ADC alone. Given the limited sample size, this subgroup analysis should be regarded as descriptive only. Although the observed pattern suggests that bpMRI-only assessment may be more vulnerable to misclassification in the post-treatment setting, this finding should be interpreted cautiously and not as definitive evidence.

## 4. Discussion

In this retrospective single-center study, we evaluated whether a biparametric MRI approach based on T2-weighted and DWI can reproduce routine mpMRI-based VI-RADS assessment in a contemporary real-world cohort, and we specifically examined the influence of radiologist expertise on agreement and clinically relevant misclassification patterns. Our findings should be interpreted within the clinical setting for which VI-RADS is most strongly supported, namely preoperative local staging before TURBT in patients with suspected or known bladder cancer. This distinction is important because VI-RADS was originally developed for this primary-staging context, whereas post-TURBT examinations are more vulnerable to inflammatory, hemorrhagic, and fibrotic changes that may artificially alter the apparent tumor–muscle interface. Accordingly, our mixed cohort should not be interpreted as implying equal robustness of bpMRI or mpMRI across all clinical scenarios [24]. The main finding is that bpMRI should not be considered a routine substitute for contrast-enhanced mpMRI within a comprehensive VI-RADS workflow. Agreement with the reference mpMRI report was strongly reader-dependent, with higher concordance for the experienced genitourinary radiologist than for the non-expert reader, and this difference was clinically relevant because bpMRI-related discrepancies tended to cluster around decision-relevant VI-RADS boundaries rather than occurring at random.

**Figure 7:**
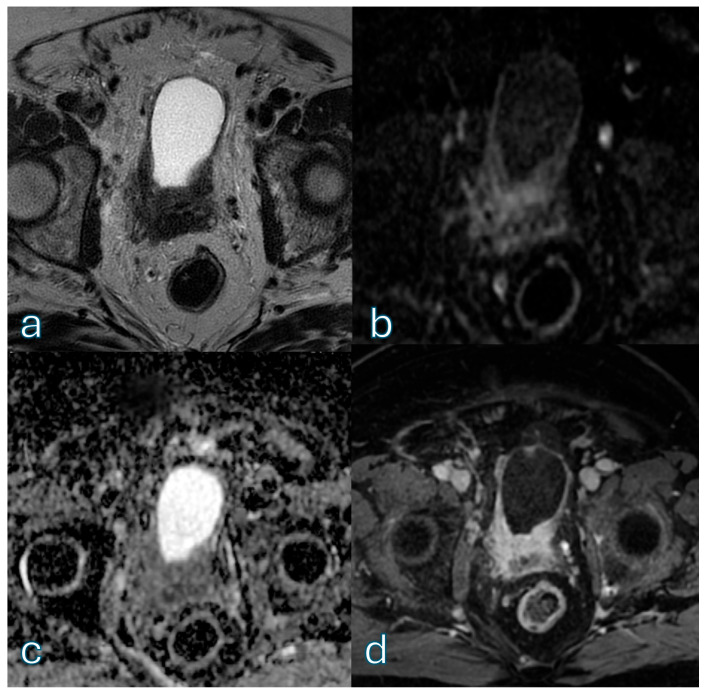
Multiparametric MRI demonstrates a lesion located at the bladder base with aggressive morphologic features. On T2-weighted imaging (**a**), there is clear disruption of the normal bladder wall layered architecture with extension beyond the muscular layer. Diffusion-weighted imaging (**b**) shows marked diffusion restriction with corresponding low signal intensity on the ADC map (**c**), consistent with high cellularity. Dynamic contrast-enhanced (**d**) imaging demonstrates early tumoral enhancement with transmural extension and extravesical spread, confirming definite muscular invasion.

Beyond global summary measures, confusion matrix analysis provided important insight into where bpMRI diverges from mpMRI-driven classification. This was also supported by the stratified disagreement analysis, which showed higher discordance in reference high-risk lesions (VI-RADS 4–5) than in low-risk lesions (VI-RADS 2–3) for both readers. By contrast, stratification by clinical setting suggested that post-TURBT-related disagreement was more pronounced for the non-expert reader, whereas conclusions for the expert reader remain limited by the very small number of post-TURBT cases. In particular, discrepancies were common at higher-risk boundaries, and shifts between VI-RADS 4 and 5 occurred more frequently for the non-expert reader than for the expert reader. This pattern matters in practice because upper-category distinctions often influence the perceived extent of invasion and the confidence of staging, especially when MRI findings are integrated into multidisciplinary discussions. An example of definite muscular invasion with extravesical spread (high-risk category) is shown in Figure 7.

**Figure 8:**
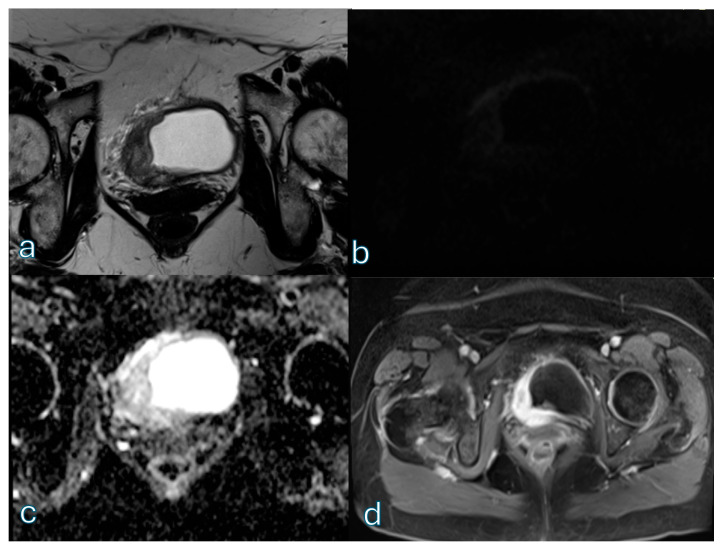
T2w Multiparametric MRI (**a**) shows a lesion arising from the right lateral bladder wall with morphologic features suspicious for high-risk disease. On diffusion-weighted imaging (DWI, (**b**)), the lesion does not demonstrate significant diffusion restriction, with no corresponding low signal on the ADC map (**c** ), a finding that could potentially lead to underestimation or misclassification. In contrast, dynamic contrast-enhanced (**d**) imaging demonstrates early enhancement with interruption of the muscularis propria without extravesical extension, allowing accurate classification as VI-RADS 4.

From a practical standpoint, our data support a bpMRI-only pathway only when all of the following conditions are met: pre-TURBT setting, no prior local treatment, adequate bladder distension and image quality, and clearly low-risk, non-equivocal findings on T2WI and DWI/ADC. Conversely, VI-RADS 3 or otherwise equivocal lesions, suspected VI-RADS ≥ 4 lesions, post-TURBT examinations, and technically suboptimal studies should remain in the standard mpMRI pathway with DCE. This distinction is clinically relevant because, at the VI-RADS ≥ 4 threshold, both readers maintained high sensitivity, but the non-expert reader showed lower specificity, consistent with a greater tendency to overstage lesions without the stabilizing contribution of contrast-enhanced imaging. In practical terms, overstaging may increase unnecessary aggressive workup, strengthen suspicion of muscle-invasive disease during multidisciplinary discussion, and potentially influence surgical planning or treatment escalation. Based on the proportion of reference-negative lesions in our cohort, the observed specificity values translate into approximately 7 false-positive classifications per 100 similar cases for the expert reader and 13 per 100 similar cases for the non-expert reader. From an operational standpoint, these findings suggest that bpMRI interpretation is less “forgiving” than mpMRI and may demand stricter adherence to VI-RADS criteria and greater familiarity with subtle morphological and diffusion-weighted cues at the tumor–muscle interface. The absence of DCE likely contributes to these differences by removing a sequence that can clarify equivocal interfaces and reduce interpretative uncertainty. Figure 8 shows an example in which diffusion-weighted imaging is not decisive, while contrast-enhanced imaging demonstrates interruption of the muscular layer, supporting a high-risk classification.

**Figure 9:**
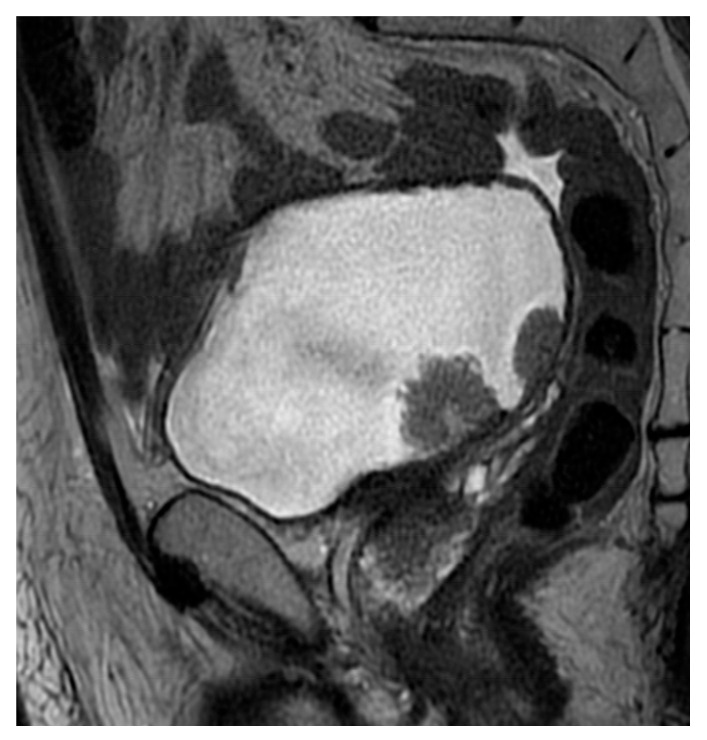
T2w sagittal Multiparametric MRI in a 70-year-old patient demonstrates two synchronous bladder lesions with distinct morphologic and functional characteristics.

**Figure 10:**
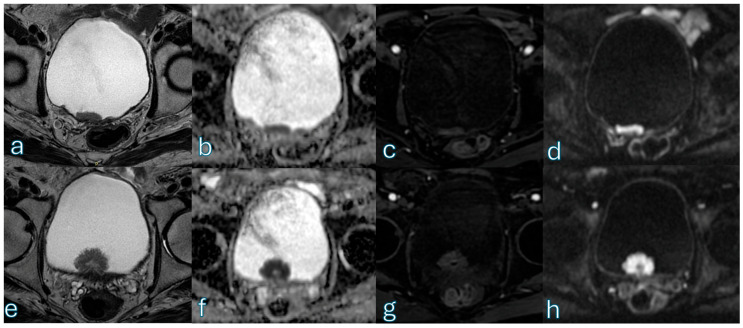
Multiparametric MRI in a 70-year-old patient demonstrates two synchronous bladder lesions with distinct morphologic and functional characteristics. The first lesion (**a**–**d**) is a flat bladder wall lesion, measuring approximately 1 cm, with equivocal findings on T2-weighted imaging (T2WI-(**a**)) and DWI/ADC (**b**,**c**). In this case, dynamic contrast-enhanced (**d**) imaging demonstrates early enhancement extending toward the muscular layer, which was fundamental for lesion characterization and supports classification as VI-RADS 3. The second lesion (**e**–**h**) is a pedunculated mass arising from the bladder wall, measuring approximately 2 cm, showing the characteristic inchworm sign on diffusion-weighted imaging (**f**) with corresponding findings on the ADC map (**g**), orienting the assessment toward a VI-RADS 2 category despite its size.

DCE provides temporal information on lesion enhancement relative to the muscularis propria and can be particularly helpful when tumor margins are irregular, when diffusion restriction is heterogeneous, or when reactive wall changes make the muscular layer difficult to delineate. An illustrative case with two synchronous lesions (Figure 9), including one equivocal lesion in which contrast-enhanced imaging was decisive for classification, is shown in Figure 10.

In a bpMRI-only setting, readers must rely predominantly on indirect anatomical cues on T2WI and on the quality and interpretability of DWI/ADC, both of which may be affected by artifacts and may be challenging in certain lesion morphologies. In this sense, DCE may act not only as an incremental imaging component, but also as a practical “equalizer” that reduces experience-related variability and narrows the interpretive gap between readers. Collectively, our results support a selective rather than universal adoption of bpMRI, emphasizing that omission of contrast is most defensible when supported by reader expertise and by a clinical scenario in which the probability of muscle invasion is clearly low.

The post-treatment setting represents a particularly critical scenario. More broadly, the question of when VI-RADS should be used is still evolving. At present, the strongest evidence supports its use for primary local staging and muscle invasion risk stratification before TURBT or before decisions regarding repeat resection and treatment planning. Emerging applications in recurrent disease, postoperative assessment, and treatment-response evaluation after systemic therapy are increasingly being explored, including nacVI-RADS, but these should be regarded as scenario-specific extensions rather than equivalent uses of the original score. In our cohort, cases acquired after TURBT showed a numerically higher frequency of bpMRI–mpMRI discrepancies for both readers, with most discordant cases reflecting overstaging on bpMRI. However, because only nine post-TURBT cases were included, this observation should be interpreted as a descriptive trend rather than as a robust subgroup effect. This trend is biologically and radiologically plausible, as postoperative inflammation, edema, granulation tissue, and fibrosis can alter bladder wall thickness and produce diffusion restriction that mimics residual or recurrent tumor on T2WI and DWI alone. In these circumstances, DCE may contribute relevant information by helping differentiate enhancing viable tumor from postoperative changes, thereby reducing interpretative ambiguity. Although the post-TURBT subgroup was small and our analysis was descriptive, the observed pattern supports caution when considering bpMRI-only assessment in post-treatment evaluations.

From a practical perspective, no dedicated biparametric protocol has yet been fully validated to replace perfusion imaging in the post-TURBT setting. Nevertheless, some measures may help reduce overstaging when a contrast-free examination is the only feasible option. MRI should ideally be performed before TURBT or at least 2 weeks after TURBT, bladder biopsy, or intravesical treatment, in order to limit reactive edema and inflammatory change. In addition, optimal bladder distension and antiperistaltic preparation remain essential to improve delineation of the bladder wall. In a bpMRI-only workflow, interpretation should rely predominantly on high-quality DWI and systematic assessment of ADC maps, because postoperative edema may generate hyperintensity related to T2 shine-through, whereas fibrosis and other benign post-manipulation changes usually do not show marked true diffusion restriction. Even so, these strategies should be regarded as mitigation measures rather than a replacement for DCE, and equivocal or post-treatment examinations should still preferentially undergo standard contrast-enhanced mpMRI whenever clinically feasible.

Several methodological aspects should be considered when interpreting our findings. First, two different reference standards were used according to the study objectives. Routine mpMRI-based VI-RADS reporting served as the reference standard for the reproducibility analysis, because the aim was to determine whether bpMRI could reproduce the standard imaging workflow used in clinical practice. By contrast, histopathology served as the reference standard for the binary diagnostic endpoint of muscle-invasive versus non-muscle-invasive disease. This dual-reference-standard design reflects both the practical clinical question of protocol interchangeability and the biological gold standard for muscle invasion. At the same time, histopathology does not provide a direct one-to-one counterpart for the full ordinal VI-RADS scale; therefore, categorical agreement analyses necessarily relied on the imaging-based mpMRI reference. Accordingly, the present findings should be interpreted primarily as evidence of reproducibility relative to routine mpMRI-based VI-RADS reporting within a real-world institutional setting, rather than as a direct pathology-validated assessment of muscle invasion staging performance. Second, the study was retrospective and single-center, and the sample size was moderate, limiting the precision of subgroup observations. In addition, although bpMRI cases were anonymized and reviewed in randomized order by readers who were different from the original mpMRI reporting radiologist, the study did not adopt a fully prospective paired-reading design in which the same examinations are interpreted under both bpMRI and mpMRI conditions according to a randomized sequence. Therefore, some residual reading-order or design-related bias cannot be completely excluded. This limitation should be considered when comparing our results with more controlled multireader paradigms developed in other MRI reporting systems. Third, the lesion-based design introduces potential within-patient correlation in cases with multiple lesions. Although lesions were scored independently and lesion-specific staging of one lesion did not determine the score of another, they still shared the same patient-level and examination-level context; therefore, statistical independence cannot be fully assumed. In our cohort, however, clustering was limited, since 61 patients had one lesion and only 4 patients had two lesions. We therefore believe that the magnitude of this effect was modest, but confidence intervals may still be somewhat optimistic and results should be interpreted accordingly. Finally, generalizability may be influenced by local acquisition protocols and by the VI-RADS distribution of the cohort, which included a substantial proportion of intermediate- and high-risk lesions typical of a tertiary referral population.

Despite these limitations, the study has strengths that support the robustness and practical relevance of its message. Notably, the inclusion of one dedicated genitourinary radiologist and one non-expert reader was intentional and reflected the specific study aim of assessing the impact of reader experience on bpMRI reliability. Accordingly, this aspect should be interpreted as a design feature rather than as unintended reader-selection bias, although it may limit generalizability to other reader populations. The design reflects a realistic scenario in which bpMRI is considered as a contrast-free alternative, and the direct comparison between an expert and a non-expert reader provides actionable insight into how reader experience modifies bpMRI reliability. In addition, the combined use of weighted kappa, confusion matrices, and ROC analysis allowed us to move beyond single summary metrics and to describe where and how bpMRI diverges from mpMRI-based VI-RADS assessment, including at clinically meaningful thresholds.

Overall, our findings support an expertise- and context-dependent use of bpMRI for bladder cancer assessment. The present results should be interpreted in light of both study aims: bpMRI showed reader-dependent reproducibility relative to standard mpMRI VI-RADS reporting and also demonstrated good diagnostic concordance with histopathology for the binary endpoint of muscle invasion. bpMRI can contribute to streamlined imaging pathways only in a restricted clinical scenario, namely treatment-naïve pre-TURBT patients with adequate image quality and clearly low-risk, non-equivocal findings on T2WI and DWI/ADC. By contrast, equivocal lesions, suspected intermediate- or high-risk disease, post-TURBT examinations, and technically limited studies should still undergo standard contrast-enhanced mpMRI to ensure more reliable and reproducible VI-RADS staging. Future multicenter studies with standardized acquisition parameters and histopathological correlation, as well as evaluations of structured training interventions, will be important to clarify how bpMRI can be safely integrated into clinical workflows and which patient subgroups derive the greatest benefit from a contrast-free approach.

## 5. Conclusions

Biparametric MRI should not be considered a routine replacement for contrast-enhanced multiparametric MRI for comprehensive VI-RADS assessment. While bpMRI may provide acceptable mpMRI-consistent risk stratification when interpreted by experienced radiologists in treatment-naïve pre-TURBT patients with clearly low-risk, non-equivocal imaging findings, its reliability appears lower in equivocal lesions, intermediate- and high-risk disease, and in the post-TURBT setting, with greater variability in less experienced readers. The latter observation should be interpreted cautiously because the post-TURBT subgroup in our cohort was small. In post-TURBT patients, if contrast administration is not feasible, a carefully optimized bpMRI examination with strict bladder preparation and DWI/ADC-focused interpretation may reduce misclassification, but it should not be considered equivalent to standard mpMRI in equivocal cases. Clinical context and radiologist experience remain key determinants of bpMRI safety and effectiveness, and mpMRI continues to represent the reference approach for oncologic bladder MRI in routine practice, particularly in the primary pre-TURBT staging setting for which VI-RADS is best established. Post-TURBT and other post-treatment scenarios remain more challenging and should be approached with greater caution, especially when contrast-enhanced imaging is omitted.

## Figures and Tables

**Table 1:** Patient demographics and clinical characteristics.

Variable	Value
Number of patients (*n*)	65
Number of lesions (*n*)	69
Male/Female (*n*)	47/18
Age (years, range)	65–83
Priot TURBT (*n*, %)	9 (13.8%)
MRI period (month, year)	Jan 2024–Dec 2024

**Table 2:** Confusion matrix for bpMRI-based VI-RADS assessment by the expert reader.

mpMRI vs. bpMRI	Vi-Rads 2	Vi-Rads 3	Vi-Rads 4	Vi-Rads 5
Vi-Rads 2 (*n*)	21	0	0	3
Vi-Rads 3 (*n*)	1	7	2	0
Vi-Rads 4 (*n*)	2	1	8	3
Vi-Rads 5 (*n*)	1	0	5	15

**Table 3:** Confusion matrix for bpMRI-based VI-RADS assessment by the non-expert reader.

mpMRI vs. bpMRI	Vi-Rads 2	Vi-Rads 3	Vi-Rads 4	Vi-Rads 5
Vi-Rads 2 (*n*)	17	1	1	5
Vi-Rads 3 (*n*)	2	5	1	2
Vi-Rads 4 (*n*)	0	2	6	6
Vi-Rads 5 (*n*)	1	1	8	11

**Table 4:** Agreement and discrimination metrics.

Metric	Expert Reader	Non-Expert Reader
Weighted Cohen’s κ (quadratic)	0.74 (0.56–0.89)	0.58 (0.37–0.75)
AUC for Vi-Rads ≥ 4	0.87 (0.78–0.95)	0.81 (0.69–0.91)
Overall Agreement (%)	73.9%	56.5%

**Table 5:** Distribution of concordant, overstaged, and understaged bpMRI classifications relative to the reference mpMRI report in the post-TURBT subgroup.

Reader	Concordant	Overstaged	Understaged	Total
Expert	8	1	0	9
Non expert	5	3	1	9

## Data Availability

The data presented in this study are available on request from the corresponding author.
